# Exact analytical solutions of the Bloch equation for the hyperbolic‐secant and chirp pulses

**DOI:** 10.1002/mrm.30603

**Published:** 2025-06-16

**Authors:** Ryan H. B. Smith, Donald Garwood, Michael Garwood

**Affiliations:** ^1^ Department of Radiation Oncology University of Minnesota School of Medicine Minneapolis Minnesota USA; ^2^ Center for Magnetic Resonance Research and Department of Radiology University of Minnesota Minneapolis Minnesota USA

**Keywords:** analytical solution, BIR‐4, Bloch equation, chirp, hyperbolic‐secant, radiofrequency pulse

## Abstract

**Purpose:**

To improve the accuracy and generality of analytical solutions of the Bloch equation for the hyperbolic‐secant (HS1) and chirp pulses in order to facilitate application to truncated and composite pulses and use in quantitative methods.

**Theory and Methods:**

Previous analytical solutions of the Bloch equation during an HS1 pulse driving function are refined and extended in this exact solution for arbitrary initial magnetization and pulse parameters including asymmetrical truncation. An unapproximated general solution during the chirp pulse is derived in a non‐spinor formulation for the first time. The solution on the extended complex plane for the square pulse is included for completeness.

**Results:**

The exact solutions for the HS1, chirp, and square pulses demonstrate high consistency with Runge‐Kutta simulations for all included pulse and isochromat parameters. The HS1 solution is strictly more accurate than the most complete prior general solution. The analytical solution of the BIR‐4 composite pulse constructed using asymmetrically truncated HS1 component pulses likewise agrees with simulation results.

**Conclusion:**

The derived analytical solutions for the Bloch equation during an HS1 or chirp pulse are exact regardless of pulse parameters and initial magnetization and precisely conform with simulations enabling their use in quantitative MRI applications and setting a foundation for the analytical consideration of relaxation and pulses in multiply rotating frames.

## 1 INTRODUCTION

Frequency‐modulated (FM) pulses are often exploited in demanding NMR and MRI methods due to their ability to produce high quality, reproducible data. For instance, they are commonly implemented in pulse sequences that benefit from having resilience to spatially‐nonuniform $B_{1}$ and $B_{0}$ amplitudes. For such purposes, the hyperbolic secant pulse (HS1),^1^, ^2^ which has its origins in optical resonance^3^, remains one of the oldest and most popular pulses. The HS1 pulse is based on amplitude‐modulated (AM) and FM functions of the hyperbolic secant (sech) and hyperbolic tangent (tanh), respectively. Another FM pulse that enjoys wide utility in NMR and MRI is the chirp pulse that originated in the field of radar. Chirp has constant RF amplitude and a linear frequency sweep. Chirp is commonly exploited in NMR and MRI applications that require broadband spin manipulations. The latter can be difficult to achieve with conventional, constant frequency pulses due to the inverse relationship between pulse length ($T_{p}$) and bandwidth. With any such pulse, the maximum attainable RF amplitude is usually a practical barrier to exciting large bandwidths.

In the past, many other pairs of AM and FM functions have been introduced and shown to perform as well as or better than HS1 and chirp pulses under specific experimental conditions. Superior experimental precision and robustness are indeed possible with other AM/FM pulse pairs, but it can be argued that this is only true when the pulse is judged by a limited set of performance criteria. The HS1 pulse is generally unmatched when considering a broad range of performance specifications simultaneously, such as the uniformity of flip angle, the sharpness of the baseband demarcation, and invariance to large $B_{1}$ and $B_{0}$ inhomogeneity. Despite efforts to derive improved AM/FM pairs, the HS1 pulse generally remains the gold standard for comparing the performance of different pulses.

As a result of extensive study of the HS1 driving function originally in optical resonance and later in magnetic resonance research, many unique properties of the sech/tanh pair of functions have been identified and are now well understood. In optical spectroscopy and magnetic resonance, an analysis of vector trajectories of the dipole and magnetic moments generally requires numerical solutions of the (nonlinear) Bloch equation. The lack of analytical solutions to describe magnetization trajectories of spin‐1/2 isochromats produced by some pulses has been less than ideal for certain advanced purposes, including efforts to extend formalisms used to describe adiabatic rotating frame relaxation^4^, ^5^, ^6^, ^7^ and to employ model‐based image reconstruction approaches.^8^, ^9^, ^10^ However, the HS1 pulse holds advantages in this regard as well. An exact analytical solution for the specific case of the HS1 pulse that has a frequency sweep amplitude (A) equal to its maximum Rabi frequency ($\omega_{1}^{m}$) was first described by Allen and Eberly.^11^ For the case of zero frequency modulation (A=0), McCall and Hahn analytically described a phenomenon called self‐induced transparency (SIT) in which the magnetization trajectory produced by an HS1 pulse with area 2π executes a perfect rotation back to its initial polarized orientation along the z‐axis regardless of resonance offset.^3^ Analytical solutions for the HS1 pulse under more general conditions have also been derived^1^, ^2^, ^12^, ^13^, ^14^ with similar spinor formulation derivations for chirp.^15^, ^16^ The analytic solution for the HS1 pulse by Zhang et al.^13^ extended previous work through robust treatment of transverse magnetization and arbitrary initial magnetization orientation but relied on assumptions valid only in the limit of low truncation to define particular solutions. These assumptions limit accuracy at low truncations and prevent application to cases in which the truncation is large, such as in composite FM pulses like the $B_{1}$‐insensitive rotation pulse 4 (BIR‐4).^17^ In this paper, we complete the previous solutions of the Bloch equation for the HS1 pulse by presenting the exact solution with and without FM regardless of truncation. We then apply this mathematical approach to derive the exact solution for the chirp pulse from the Bloch‐Riccati equation. The extended complex plane solution to the square pulse, previously derived in standard coordinates,^18^ is included for completeness.

## 2 THEORY

### 2.1 General approach using the Bloch‐Riccati equation

Any RF pulse $\omega_{1}$ applied in the transverse plane of the phase‐modulated rotating frame at $\omega_{c}$ can be written in terms of its AM and FM functions ($\omega_{\text{AM}}$ and $\omega_{\text{FM}}$, respectively) as

(1) $$\begin{array}{ll} \omega_{1}(t) & =\omega_{\text{AM}}(t)e^{i\phi_{1}},\\ \phi_{1}(t) & =\int \left(\omega_{c}-\omega_{\text{FM}}(t^{\prime })\right)dt^{\prime } \end{array}$$

where $\phi_{1}$ is the phase of the applied RF field. The Bloch equation neglecting relaxation during any such pulse in the same rotating frame is

(2) $$\frac{dM}{dt}=\left[\begin{array}{lll} 0 & \Omega & -\omega_{1y}\\ -\;\Omega & 0 & \omega_{1x}\\ \omega_{1y} & -\omega_{1x} & 0 \end{array}\right]M$$

with resonance offset $\Omega =\omega_{0}-\omega_{c}$. This equation can be reformulated through stereographic projection of the magnetization to the Riemann sphere. This projection and its inverse are

(3) $$\begin{array}{ll} f & =\frac{M_{x}+iM_{y}}{|M|+M_{z}} \end{array}$$

(4) $$\begin{array}{ll} M_{x}+iM_{y} & =|M|\frac{2f}{1+|f{|}^{2}},\;M_{z}=|M|\frac{1-|f{|}^{2}}{1+|f{|}^{2}}. \end{array}$$

In MRI and optical resonance literature, this projection and its inverse are usually defined at equilibrium magnetization (i.e., $|M|=M_{0}$); however, they represent a bijective relationship preserving physical meaning for any $\left|M|\in (0,M_{0}\right]$. Applying this projection to Equation (2) yields the Bloch‐Riccati equation

(5) $$\frac{df}{dt}=\frac{i}{2}\omega_{1}(t)-i\Omega f-\frac{i}{2}\omega_{1}^{*}(t)f^{2}.$$

### 2.2 General solution for the HS1 pulse

The AM and FM functions for the HS1 pulse with duration $T_{p}$ centered on the domain $t\in [0,T_{p}]$ can be written as

(6) $$\begin{array}{ll} \omega_{\text{AM}}(t) & =\omega_{1}^{m}\text{sech}\left(\frac{2\beta }{T_{p}}\left(t-\frac{T_{p}}{2}\right)\right),\\ \omega_{\text{FM}}(t) & =A\tanh\left(\frac{2\beta }{T_{p}}\left(t-\frac{T_{p}}{2}\right)\right) \end{array}$$

where β is a unitless truncation factor and A is clarified to be the frequency sweep magnitude in the limit of negligible truncation. β is defined such that sech(β) is the fractional truncation amplitude of $\omega_{\text{AM}}$ at the pulse boundaries. The sign of β controls the direction of the frequency sweep. For truncated pulses, the actual frequency sweep magnitude is decreased by a factor of tanhβ with a corrected time‐bandwidth factor

(7) $$R=\frac{AT_{p}}{\pi }\tanh(\beta )$$

which approximately equals $AT_{p}/\pi$ if truncation is small. The pulse phase can then be derived as

(8) $$\begin{array}{ll} \phi_{1}(t) & =A\int_{T_{p}/2}^{t}\tanh\left(\frac{2\beta }{T_{p}}\left(t^{\prime }-\frac{T_{p}}{2}\right)\right)\;dt^{\prime }\\ & =\frac{AT_{p}}{2\beta }\ln\left|\frac{\omega_{1}^{m}}{\omega_{\text{AM}}(t)}\right|. \end{array}$$

**FIGURE 1:**
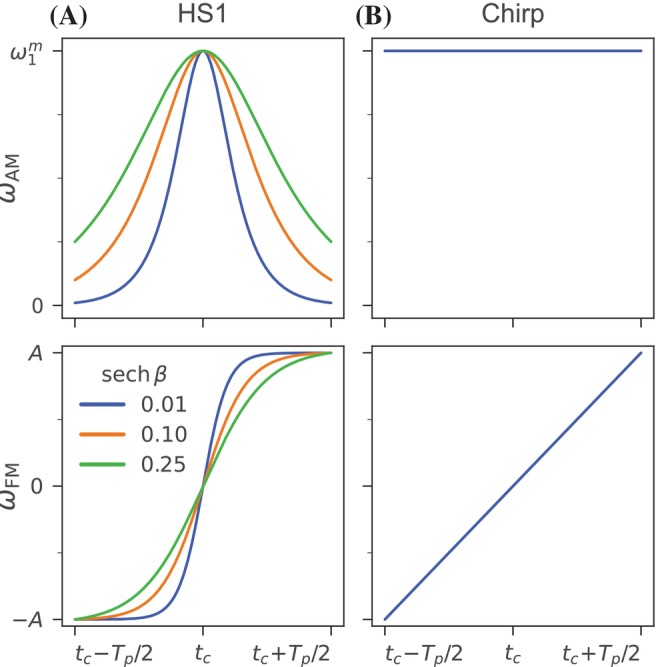
Labeled pulse diagrams for the (A) HS1 and (B) chirp pulses showing the AM and FM functions (respectively $\omega_{\text{AM}}$ and $\omega_{\text{FM}}$). The shapes of the HS1 AM and FM functions depend on the fractional truncation as specified by β and are shown for three representative symmetrical truncations.

These definitions are convenient and can represent any symmetrically truncated pulse through transformation of the time domain (if pulse onset is at t≠0) and phase (if $\phi_{1}(T_{p}/2)\neq 0$). To avoid unnecessary variable transformations and precisely define parameters for asymmetrically truncated pulses, we instead define generalized AM, FM, and phase functions for all t as

(9) $$\begin{array}{ll} \omega_{\text{AM}}(t) & =\omega_{1}^{m}\text{sech}\left(\frac{2\beta }{T_{p}}\left(t-t_{c}\right)\right),\\ \omega_{\text{FM}}(t) & =A\tanh\left(\frac{2\beta }{T_{p}}\left(t-t_{c}\right)\right),\\ \phi_{1}(t) & =\phi_{c}+\frac{AT_{p}}{2\beta }\ln\left|\frac{\omega_{1}^{m}}{\omega_{\text{AM}}(t)}\right| \end{array}$$

where $t_{c}$ and $\phi_{c}$ are, respectively, the time and pulse phase corresponding to the peak amplitude of $\omega_{\text{AM}}$ and where $T_{p}$ is more precisely defined as twice the time interval from $t_{c}$ at which β determines the fractional value of $\omega_{\text{AM}}$ (i.e., $\omega_{\text{AM}}(t_{c}\pm T_{p}/2)/\omega_{1}^{m}=\text{sech}(\beta )$). For clarity, Figure 1A shows the HS1 AM and FM functions labeled under this convention.

With this generalized pulse definition, the nonlinear Equation (5) can be transformed into a linear hypergeometric differential equation through the introduction of the two new variables

(10) $$\begin{array}{ll} p & =\frac{1}{2}\left(1+\tanh\left(\frac{2\beta }{T_{p}}\left(t-t_{c}\right)\right)\right), \end{array}$$

(11) $$\begin{array}{ll} \ln q & =\frac{i}{2}\int \omega_{1}^{*}(t^{\prime })f(t^{\prime })\;dt^{\prime } \end{array}$$

in which q is intentionally defined using the indefinite integral. Equation (5) can be transformed using these variable definitions into

(12) $$\begin{array}{ll} & p(1-p)\frac{d^{2}q}{dp^{2}}+\left(\frac{1}{2}+i\frac{T_{p}}{4\beta }(\Omega +A)-\left(1+i\frac{T_{p}}{2\beta }A\right)p\right)\frac{dq}{dp}\\ & \;+{\left(\frac{\omega_{1}^{m}T_{p}}{4\beta }\right)}^{2}q=0 \end{array}$$

for which a sufficiently general solution and its derivative written in terms of its special solutions for p near 0 are

(13) $$\begin{array}{ll} q & =F_{A}(p)+CF_{B}(p),\\ \frac{dq}{dp} & =F_{A}^{\left(1\right)}(p)+CF_{B}^{\left(1\right)}(p) \end{array}$$

with 

(14)

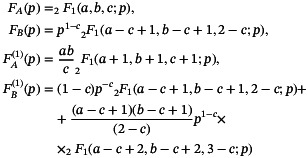


where 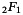 is the Gauss hypergeometric function and the constants a, b, and c are defined as

(15) $$\begin{array}{ll} a & =\frac{T_{p}}{4\beta }\left(iA+\sqrt{{\left(\omega_{1}^{m}\right)}^{2}-A^{2}}\right),\\ b & =\frac{T_{p}}{4\beta }\left(iA-\sqrt{{\left(\omega_{1}^{m}\right)}^{2}-A^{2}}\right),\\ c & =\frac{1}{2}+i\frac{T_{p}}{4\beta }\left(\Omega +A\right) \end{array}$$

which in the case of no FM (i.e., A=0), simplify to

(16) $$a=\frac{T_{p}\omega_{1}^{m}}{4\beta },\;b=-\frac{T_{p}\omega_{1}^{m}}{4\beta },\;c=\frac{1}{2}+i\frac{T_{p}\Omega }{4\beta }.$$

Substitution of Equation (13) into Equation (11) results in the expression for the general solution of the Bloch‐Riccati equation (Equation 5) subject to an HS1 driving function:

(17) $$\begin{array}{ll} f & =-i\frac{2\beta \omega_{1}(p)}{T_{p}{\left(\omega_{1}^{m}\right)}^{2}}\frac{1}{q}\frac{dq}{dp}\\ & =-i\frac{2\beta \omega_{1}(p)}{T_{p}{\left(\omega_{1}^{m}\right)}^{2}}\left(\frac{F_{A}^{\left(1\right)}(p)+CF_{B}^{\left(1\right)}(p)}{F_{A}(p)+CF_{B}(p)}\right). \end{array}$$

From this result, the transverse and longitudinal magnetization components can be determined through inverse stereographic projection (Equation 4).

### 2.3 Exact particular solution for the HS1 pulse

This presented general solution expands on the previous solution by Zhang et al.^13^ through inclusion of generalizations to the magnetization projection and pulse definitions and inclusion of explicit definitions for a, b, and c in the case of no FM (Equation 16). However, the most significant difference is the simplification of the general solution for f (Equation 17) to only include a single constant C. This is equivalent to the previous two constant solutions (using $C_{A}$ and $C_{B}$) by the following derivation:

(18) $$\begin{array}{ll} f & =-i\frac{2\beta \omega_{1}(p)}{T_{p}{\left(\omega_{1}^{m}\right)}^{2}}\left(\frac{C_{A}F_{A}^{\left(1\right)}(p)+C_{B}F_{B}^{\left(1\right)}(p)}{C_{A}F_{A}(p)+C_{B}F_{B}(p)}\right)\\ & =-i\frac{2\beta \omega_{1}(p)}{T_{p}{\left(\omega_{1}^{m}\right)}^{2}}\left(\frac{C_{A}}{C_{A}}\right)\left(\frac{F_{A}^{\left(1\right)}(p)+\frac{C_{B}}{C_{A}}F_{B}^{\left(1\right)}(p)}{F_{A}(p)+\frac{C_{B}}{C_{A}}F_{B}(p)}\right)\\ & =-i\frac{2\beta \omega_{1}(p)}{T_{p}{\left(\omega_{1}^{m}\right)}^{2}}\left(\frac{F_{A}^{\left(1\right)}(p)+CF_{B}^{\left(1\right)}(p)}{F_{A}(p)+CF_{B}(p)}\right). \end{array}$$

The general solution for the second order Equation (12) does indeed require two constants, $C_{A}$ and $C_{B}$ and consequently two boundary conditions to specify a particular solution. However, the actual equation of interest, the first order Equation (5), mathematically requires only a single boundary condition to specify the single constant ($C=C_{B}/C_{A}$) associated with a particular solution consistent with the deterministic evolution of magnetization from any single defined point. The imposition of meaning on the value of q by defining it with a definite integral unnecessarily complicates constant determination by introducing dependence on integrals that lack analytical forms. Solving Equation (11) for the lone constant C yields

(19) $$C=-\left(\frac{f(p)F_{A}(p)+i\frac{2\beta \omega_{1}(p)}{T_{p}{\left(\omega_{1}^{m}\right)}^{2}}F_{A}^{\left(1\right)}(p)}{f(p)F_{B}(p)+i\frac{2\beta \omega_{1}(p)}{T_{p}{\left(\omega_{1}^{m}\right)}^{2}}F_{B}^{\left(1\right)}(p)}\right)$$

which is specified without approximation by any single known magnetization at any time p(t) during the pulse.

### 2.4 General and exact particular solutions for the square chirped pulse

The HS1 driving function approaches the chirp driving function in the limit of symmetrical complete truncation, suggesting a solution through an analogous approach differing primarily in its reparameterization of the time domain. For this approach, the chirp AM, FM, and derived phase functions can be written as

(20) $$\begin{array}{ll} \omega_{\text{AM}}(t) & =\omega_{1}^{m},\\ \omega_{\text{FM}}(t) & =\frac{2A}{T_{p}}\left(t-t_{c}\right),\\ \phi_{1}(t) & =\phi_{c}+\frac{A}{T_{p}}{\left(t-t_{c}\right)}^{2} \end{array}$$

in which $T_{p}$ is defined as the time duration over which the frequency sweep width is 2A. For clarity, Figure 1B shows the chirp AM and FM functions labeled as defined above.

Constraining A≠0, the nonlinear Equation (5) can be transformed into a linear confluent hypergeometric differential equation in terms of q as defined in Equation (11) and

(21) $$p=\sqrt{\frac{iA}{T_{p}}}\left(t-t_{c}+\frac{T_{p}\Omega }{2A}\right)$$

with the result

(22) $$\frac{d^{2}q}{dp^{2}}+2p\frac{dq}{dp}-i\frac{T_{p}{\left(\omega_{1}^{m}\right)}^{2}}{4A}q=0.$$

A sufficiently general solution and its derivative for this differential equation can be written in the form

(23) $$\begin{array}{ll} q & =\left(F_{A}(p)+CF_{B}(p)\right)e^{-p^{2}},\\ \frac{dq}{dp} & =\left(F_{A}^{\left(1\right)}(p)+CF_{B}^{\left(1\right)}(p)\right)e^{-p^{2}} \end{array}$$

with 

(24)

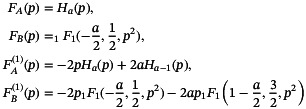


where $H_{a}$ is the Hermite polynomial of complex order a and 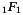 is the confluent hypergeometric function of the first kind. These functions are parameterized by a single constant

(25) $$a=-1-i\frac{T_{p}{\left(w_{1}^{m}\right)}^{2}}{8A}.$$

With these definitions, the solution to the Bloch‐Riccati equation (Equation 5) subject to a chirp driving function is given by

(26) $$\begin{array}{ll} f & =(1-i)\sqrt{\frac{2A}{T_{p}}}\frac{\omega_{1}(p)}{{\left(\omega_{1}^{m}\right)}^{2}}\frac{1}{q}\frac{dq}{dp}\\ & =(1-i)\sqrt{\frac{2A}{T_{p}}}\frac{\omega_{1}(p)}{{\left(\omega_{1}^{m}\right)}^{2}}\left(\frac{F_{A}^{\left(1\right)}(p)+CF_{B}^{\left(1\right)}(p)}{F_{A}(p)+CF_{B}(p)}\right) \end{array}$$

for which the particular solutions are specified without approximation by a single constant determined through evaluation of Equation (11) as

(27) $$C=-\left(\frac{f(p)F_{A}(p)+(i-1)\sqrt{\frac{2A}{T_{p}}}\frac{\omega_{1}(p)}{{\left(\omega_{1}^{m}\right)}^{2}}F_{A}^{\left(1\right)}(p)}{f(p)F_{B}(p)+(i-1)\sqrt{\frac{2A}{T_{p}}}\frac{\omega_{1}(p)}{{\left(\omega_{1}^{m}\right)}^{2}}F_{B}^{\left(1\right)}(p)}\right).$$

### 2.5 General and exact particular solutions for the square pulse

The square chirp pulse solution above is only valid in the case of frequency modulation (i.e., A≠0). For the square pulse (i.e., A=0) written as

(28) $$\begin{array}{ll} \omega_{\text{AM}}(t) & =\omega_{1}^{m},\\ \phi_{1}(t) & =\phi_{c}, \end{array}$$

Equation (5) has a general solution of the form

(29) $$f=\frac{e^{i\phi_{c}}}{\omega_{1}^{m}}\left(-\Omega +\sqrt{\Omega^{2}+{\left(\omega_{1}^{m}\right)}^{2}}\tanh\left(C+\frac{i}{2}t\sqrt{\Omega^{2}+{\left(\omega_{1}^{m}\right)}^{2}}\right)\right).$$

The particular solution corresponding to an isochromat with orientation $f_{c}$ at time $t_{c}$ is specified without approximation by the constant

(30) $$C=\tanh^{-1}\left(\frac{\omega_{1}^{m}e^{i\phi_{c}}f_{c}+\Omega }{\sqrt{\Omega^{2}+{\left(\omega_{1}^{m}\right)}^{2}}}\right)-\frac{i}{2}t_{c}\sqrt{\Omega^{2}+{\left(\omega_{1}^{m}\right)}^{2}}.$$

## 3 METHODS

All simulations were performed using a classic Runge‐Kutta (RK4) numerical simulation. Non‐trivial analytical calculations including hypergeometric series summations were performed with mpmath v1.3.^19^ The code written for this publication is available on GitHub.

Error in magnetization has most commonly been reported as the absolute or relative errors in the transverse and longitudinal magnetizations. These representations are intuitive and, critically, report the error in the experimentally measured transverse magnetization. However, proper interpretation can be difficult or misleading because these measures are frame dependent and exhibit relative error normalization concerns near the poles of complete longitudinal magnetization. We suggest and use an alternative symmetrical, frame‐independent error metric defined by the angle between two magnetization vectors. This metric converges with the standard unnormalized transverse error in the limit of transverse magnetization and low error while maintaining intelligibility in all cases. An example showing the relative independence of angular error on magnetic orientation is shown in Figure S1.

## 4 RESULTS

### 4.1 Simplification of the HS1 solution consistent with SIT

The exact analytical solution can be simplified for some initial conditions including the on‐resonance isochromat at equilibrium subjected to a 2π non‐frequency modulated HS1 pulse. This result has been shown as SIT in optical resonance^3^ and applied to MRI using a spinor formulation.^12^ Direct mathematical simplification of the presented solution for this particular scenario agrees with these prior results.

Consider a low truncation (i.e., arctan(expβ)≈π/2) non‐frequency modulated HS1 pulse acting on an on‐resonance isochromat initially at equilibrium. In this case C=0, and the hypergeometric parameters are

(31) $$a=\frac{T_{p}\omega_{1}^{m}}{4\beta },\;b=-a,\;c=\frac{1}{2}.$$

Substitution into Equation (17) yields 

(32)

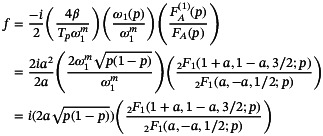


which can be simplified using the identities 

(33)

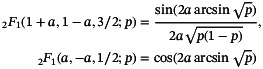


to yield

(34) $$f=i\tan\left(2a\arcsin\sqrt{p}\right).$$

The magnetization components as calculated by Equation (4) are

(35) $$\begin{array}{ll} M_{y} & =\sin\left(4a\arcsin\sqrt{p}\right),\\ M_{z} & =\cos\left(4a\arcsin\sqrt{p}\right) \end{array}$$

and the polar angle of magnetization during the pulse (after substitution for p) is

(36) $$\theta (t)=\frac{\omega_{1}^{m}T_{p}}{\beta }\arctan\left(\exp\left(\frac{2\beta }{T_{p}}(t-t_{c})\right)\right)$$

which is equivalent (ignoring constants) to the pendulum solution for an oscillation with infinite period as specified in the original proof by McCall and Hahn of self‐induced transparency for the HS1 pulse.^3^ This result can be used to calculate $\omega_{1}^{m}$ for an HS1 pulse exhibiting self‐induced transparency by observing that the final angle for an arbitrary pulse per Equation (36) is

(37) $$\theta_{f}=\frac{\omega_{1}^{m}T_{p}}{\beta }\arctan(\exp\beta )$$

from which the pulse amplitude for a 2π pulse can be derived to be

(38) $$\omega_{1}^{m}=\frac{2\pi \beta }{T_{p}\arctan(\exp\beta )}.$$

Figure S2 shows the convergence of this simple solution with simulation results at low truncation.

### 4.2 Accuracy of the HS1 analytical solution

**FIGURE 2:**
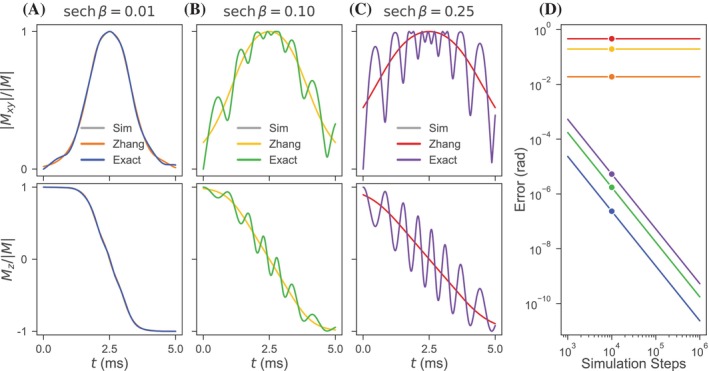
Temporal evolution of the magnetization of on‐resonance equilibrium isochromats during HS1 inversion pulses with respective pulse truncations of (A) 0.01, (B) 0.1, and (C) 0.25 as calculated using the exact analytical solution, the method of Zhang et al., and RK4 numerical simulation. The rows of the columns (A)–(C) respectively show the normalized absolute value of the transverse magnetization and the normalized longitudinal magnetization. (D) The final angular error of analytical solutions relative to simulation results is plotted as a function of simulation time steps. Consistent parameters for these calculations and simulations include $\omega_{1}^{m}/2\pi =2$ kHz, $T_{p}=5.0$ ms, and R=10 with the simulations of (A)–(C) calculated over 10,000 time steps.

The presented exact analytical solution for isochromats subject to an HS1 driving pulse with or without FM is mathematically valid for all pulse amplitudes, durations, truncations, and frequency sweep magnitudes and any isochromat off‐resonance and initial magnetization orientation. The temporal evolutions of an example isochromat initially at equilibrium as determined through RK4 simulation, the solution by Zhang et al., and the exact solution are shown for three pulse truncations in Figure 2A–C. Across the transverse and longitudinal magnetization plots for all three truncations, the exact solution agrees with the simulated results so closely that the plotted simulation line cannot be observed: The average angular error is less than $10^{-}5$ radians. The exact and simulated solutions show strong agreement with the Zhang et al. solution at low truncation with increasing discrepancy at increasing truncation. The final angular errors of the two analytical solutions for the three pulse truncations with respect to the simulated solution are shown in Figure 2D as a function of the simulation number of time steps. The Zhang et al. solutions demonstrate error that is numerically independent of the increasing number of simulated time steps with angular accuracy primarily determined by pulse truncation. The exact solutions show decreasing angular error with finer temporal discretization approaching working machine precision. In addition to varying the pulse truncation, varying the isochromat off‐resonance, initial magnetization, pulse amplitude, pulse duration, and/or frequency sweep magnitude yields comparable results (see Figures S3 and S4).

### 4.3 Accuracy of the chirp analytical solution

**FIGURE 3:**
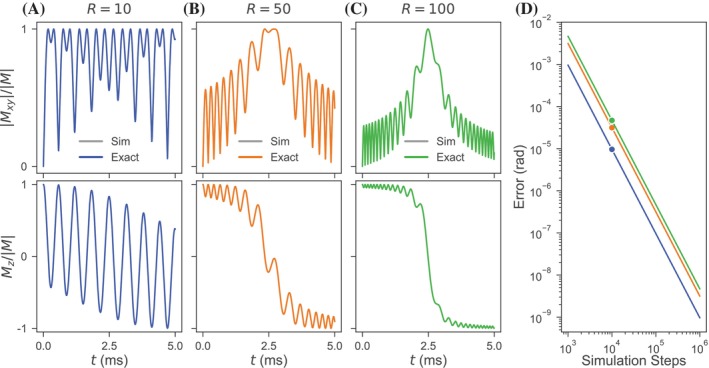
Temporal evolution of the magnetization of on‐resonance equilibrium isochromats during chirp approximate inversion pulses with respective pulse time‐bandwidth products of (A) 10, (B) 50, and (C) 100 as calculated using the exact analytical solution and RK4 numerical simulation. The rows of the columns (A)–(C) respectively show the normalized absolute value of the transverse magnetization and the normalized longitudinal magnetization. (D) The final angular error of analytical solutions relative to simulation results is plotted as a function of simulation time steps. Consistent parameters for these calculations and simulations include $\omega_{1}^{m}/2\pi =1.5$ kHz and $T_{p}=5.0$ ms with the simulations of (A)–(C) calculated over 10,000 time steps.

The presented analytical solutions for isochromats subjected to a chirp driving pulse are mathematically valid for all pulse amplitudes, durations, and frequency sweep magnitudes, as well as for any isochromat off‐resonance and initial magnetization orientation. The temporal evolutions of an example isochromat initially at equilibrium as determined through RK4 simulation and the exact solution are shown for three pulse time‐bandwidth products in Figure 3A–C. Across the transverse and longitudinal magnetization plots for the three time‐bandwidth products, the exact solution agrees with the simulated results closely enough that the plotted simulation line is completely covered by the calculated line. The final angular errors of the exact solution for the three time‐bandwidth products with respect to the simulated solution are shown in Figure 3D as a function of the simulation number of time steps. The exact solutions show decreasing angular error with finer simulation temporal discretization. Varying the time‐bandwidth product, isochromat off‐resonance, initial magnetization orientation, pulse duration, and/or frequency sweep magnitude yields similar results (see Figure S5). The solution provided for the non‐frequency‐modulated square pulse demonstrates comparable performance (see Figure S6).

### 4.4 Application of the HS1 solution to the BIR‐4 composite pulse

**FIGURE 4:**
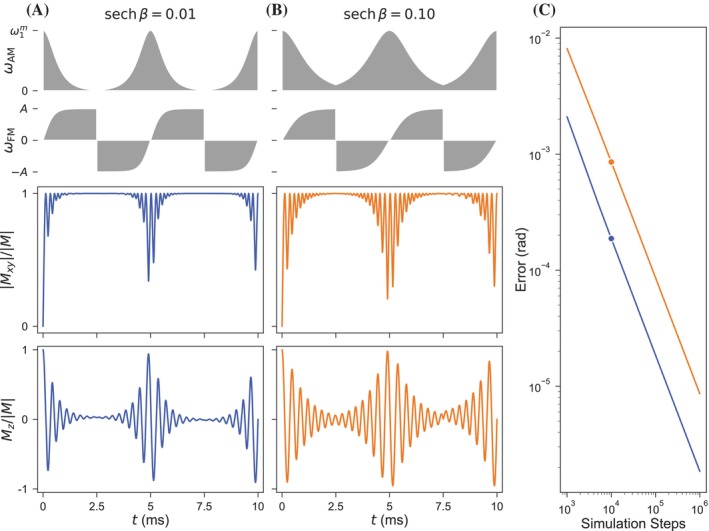
Simulated and analytically calculated temporal evolutions of the magnetization of equilibrium isochromats during composite BIR‐4 driving pulses with component pulse truncation at (A) 0.01 and (B) 0.1. The rows of the columns (A) and (B) respectively show the magnitude of $\omega_{\text{AM}}$, the magnitude of $\omega_{\text{FM}}$, the normalized absolute value of the transverse magnetization, and the normalized longitudinal magnetization. (C) The final angular error of analytical solutions relative to simulation results is plotted as a function of the total number of simulation time steps. Consistent parameters for these calculations and simulations include $\omega_{1}^{m}/2\pi =2$ kHz, Ω/2π=100 Hz, R=32, and component $T_{p}=5.0$ ms (such that the total duration of the composite BIR‐4 is 10.0 ms). The simulations of (A) and (B) were calculated over 10,000 time steps.

A particular solution of the exact HS1 analytical solution can be defined for any symmetrically or asymmetrically truncated pulse, allowing determination of magnetization orientation during composite pulses such as the BIR‐4 pulse. The BIR‐4 pulse is constructed from four asymmetrically truncated half HS1 pulses with a phase shift at the middle of the pulse that determines the final angle of excitation for an isochromat initially at equilibrium (see Figure 4 for example AM and FM functions).^17^ The temporal evolution of magnetization during two BIR‐4 excitation pulses with respective component pulse truncations of 0.01 and 0.1 as determined through the HS1 analytical solution and RK4 simulation are shown in Figure 4A,B. The plotted simulation transverse and longitudinal magnetization results are not visible because of almost exact coincidence with the plotted analytical solution. The accuracy of the analytical solution for the component HS1 pulses validates application to off resonance isochromats with initial non‐equilibrium magnetization orientation. The final angular errors of the exact solution for the two pulse truncation values with respect to the simulated solution are shown in Figure 4C as a function of the simulation number of time steps. These composite results demonstrate high accuracy and increasing concordance with an increasing number of simulation time steps.

## 5 DISCUSSION AND CONCLUSIONS

In this work we derived and evaluated exact analytical solutions for HS1 and chirp pulses. These equations precisely describe the magnetization trajectories for any resonance offset and initial magnetization orientation, regardless of pulse amplitude, duration, and frequency sweep magnitude, including no frequency sweep (i.e., A=0). Furthermore, for the case of the HS1 pulse, the equations are valid for any truncation level, which for the first time allows analytical solutions for the class of composite FM pulses like BIR‐4 that are composed of multiple asymmetrically truncated HS1 pulses.

Aside from HS1 and constant‐amplitude pulses (including chirp with A=0), most shaped RF pulses commonly used in NMR and MRI do not currently have analytical solutions. Having an analytical solution can be beneficial for speeding up the calculations of magnetization trajectories as needed, e.g., in model‐based image reconstruction and RF pulse design using physics‐guided deep learning.^20^, ^21^ Analytical solutions are theoretically capable of handling extreme parameter values that break numerical simulation; more practical benefits of analytical solutions include acting as benchmarks for simulations and in guiding theoretical pulse design. A weakness of the presented mathematics is the neglect of relaxation; however, the analytical solutions presented are a critical step toward including relaxation in the future and for extending theoretical models of rotating frame relaxation during FM pulses (e.g., adiabatic $T_{1\rho }$ and $T_{2\rho }$). Moreover, future extensions of the mathematical framework might allow other RF pulses to be described analytically. In MRI, obvious pulse candidates include the two‐ and three‐dimensional HS1 pulses^22^, ^23^, ^24^ as well as an HS1 pulse that operates in a second rotating frame of reference (e.g., for achieving a spatially‐selective spin inversion based on a $B_{1}$ gradient).

## Supporting information


**Figure S1.** The error of final magnetization between simulation and the method of Zhang et al. (respectively $M^{S}$ and $M^{C}$) shown as (a) unnormalized transverse and longitudinal error, (b) normalized transverse and longitudinal error, and (c) angular error. Initial magnetization orientation was set as M=(0,sinθ,cosθ). All calculations and simulations were performed with β=5.298, $\omega_{1}^{m}/2\pi =2$ kHz, $T_{p}=5.0$ ms, and R=8 with simulations performed over 10,000 time steps. The frequency sweep was intentionally kept low so that final magnet orientations were distributed over a range of elevations to better demonstrate the dependence of both unnormalized and normalized component error on initial (and final) orientation. Component error metrics are well behaved and accurately represent error when the meaningful error is captured in the measured plane or axis but lack the isotropism of angular error.
**Figure S2** Temporal evolution of the magnetization of on‐resonance equilibrium isochromats during HS1 inversion pulses with respective pulse truncations of (a) 0.1, (b) 0.01, and (c) 0.001 as calculated using the exact analytical solution, the SIT prediction, and RK4 numerical simulation. The rows of the columns (a)–(c) respectively show the normalized absolute value of the transverse magnetization and the normalized longitudinal magnetization. $\omega_{1}^{m}$ was determined in each case by the SIT prediction. Consistent parameters include. All results used $T_{p}=5.0$ ms and R=0 with simulations run over 10,000 time steps.
**Figure S3** Temporal evolution of the magnetization of equilibrium isochromats during HS1 pulses with Ω/2π respectively (a) 10 Hz, (b) 100 Hz, and (c) 1000 Hz as calculated using the exact analytical solution, the method of Zhang et al., and RK4 numerical simulation. The rows of the columns (a)–(c) respectively show the normalized absolute value of the transverse magnetization and the normalized longitudinal magnetization. (d) The final angular error of analytical solutions relative to simulation results is plotted as a function of simulation time steps. Consistent parameters for these calculations and simulations include β=2.993 (i.e. 10% truncation), $\omega_{1}^{m}/2\pi =2$ kHz, $T_{p}=5.0$ ms, and R=10 with the simulations of (a)–(c) calculated over 10,000 time steps.
**Figure S4** Temporal evolution of the magnetization of equilibrium isochromats during HS1 inversion pulses with R respectively (a) 5, (b) 15, and (c) 25 as calculated using the exact analytical solution, the method of Zhang et al., and RK4 numerical simulation. The rows of the columns (a)–(c) respectively show the normalized absolute value of the transverse magnetization and the normalized longitudinal magnetization. (d) The final angular error of analytical solutions relative to simulation results is plotted as a function of simulation time steps. Consistent parameters for these calculations and simulations include β=2.993 (i.e. 10% truncation), $\omega_{1}^{m}/2\pi =2$ kHz, $T_{p}=5.0$ ms, and Ω/2π=100 Hz with the simulations of (a)–(c) calculated over 10,000 time steps.
**Figure S5** Temporal evolution of the magnetization of equilibrium isochromats during chirp inversion pulses with Ω/2π respectively (a) 50 Hz, (b) 500 Hz, and (c) 5000 Hz as calculated using the exact analytical solution and RK4 numerical simulation. The rows of the columns (a)–(c) respectively show the normalized absolute value of the transverse magnetization and the normalized longitudinal magnetization. (d) The final angular error of analytical solutions relative to simulation results is plotted as a function of simulation time steps. Consistent parameters for these calculations and simulations include $\omega_{1}^{m}/2\pi =2$ kHz, $T_{p}=5.0$ ms, and R=100 with the simulations of (a)–(c) calculated over 10,000 time steps.
**Figure S6** Temporal evolution of the magnetization of equilibrium isochromats during square pulses with Ω/2π respectively (a) 0 Hz, (b) 100 Hz, and (c) 200 Hz as calculated using the exact analytical solution and RK4 numerical simulation. The rows of the columns (a)–(c) respectively show the normalized absolute value of the transverse magnetization and the normalized longitudinal magnetization. (d) The final angular error of analytical solutions relative to simulation results is plotted as a function of simulation time steps. Consistent parameters for these calculations and simulations include $\omega_{1}^{m}/2\pi =200$ Hz, $T_{p}=5.0$ ms, and $\phi_{c}=\pi /2$ with the simulations of (a)–(c) calculated over 10,000 time steps.
