# Supplementary material for: Exact analytical solutions of the Bloch equation for the hyperbolic‐secant and chirp pulses
Source: Magn Reson Med. 2025 Jun 16;94(5):2140–9. doi: 10.1002/mrm.30603 (PMC12393199; doi:10.1002/mrm.30603)
Supplement: Supplementary file 1 — Figure S1. The error of final magnetization between simulation and the method of Zhang et al. (respectively MS and MC) shown as (a) unnormalized transverse and longitudinal error, (b) normalized transverse and longitudinal error, and (c) angular error. Initial magnetization orientation was set as M=(0,sinθ,cosθ). All calculations and simulations were performed with β=5.298, ω1m/2π=2 kHz, Tp=5.0 ms, and R=8 with simulations performed over 10,000 time steps. The frequency sweep was intentionally kept low so that final magnet orientations were distributed over a range of elevations to better demonstrate the dependence of both unnormalized and normalized component error on initial (and final) orientation. Component error metrics are well behaved and accurately represent error when the meaningful error is captured in the measured plane or axis but lack the isotropism of angular error. Figure S2 Temporal evolution of the magnetization of on‐resonance equilibrium isochromats during HS1 inversion pulses with respective pulse truncations of (a) 0.1, (b) 0.01, and (c) 0.001 as calculated using the exact analytical solution, the SIT prediction, and RK4 numerical simulation. The rows of the columns (a)–(c) respectively show the normalized absolute value of the transverse magnetization and the normalized longitudinal magnetization. ω1m was determined in each case by the SIT prediction. Consistent parameters include. All results used Tp=5.0 ms and R=0 with simulations run over 10,000 time steps. Figure S3 Temporal evolution of the magnetization of equilibrium isochromats during HS1 pulses with Ω/2π respectively (a) 10 Hz, (b) 100 Hz, and (c) 1000 Hz as calculated using the exact analytical solution, the method of Zhang et al., and RK4 numerical simulation. The rows of the columns (a)–(c) respectively show the normalized absolute value of the transverse magnetization and the normalized longitudinal magnetization. (d) The final angular error of analytical solutions relative to simu [file MRM-94-2140-s001.pdf]

Supplementary Information: Exact analytical solutions of the Bloch equation for the hyperbolic-secant and chirp pulses

Ryan H. B. Smith *[1], Donald Garwood[2], and Michael Garwood[2]

[1]Department of Radiation Oncology, University of Minnesota School of Medicine, Minneapolis, MN USA
[2]Center for Magnetic Resonance Research and Department of Radiology, University of Minnesota, Minneapolis, MN USA

21 January 2025

List of Figures

S1 Comparison of transverse, longitudinal, and angular error S2
S2 HS1 solution convergence with simple SIT predictions S3
S3 Accuracy of the HS1 solution for variable Ω S4
S4 Accuracy of the HS1 solution for variable R S5
S5 Accuracy of the chirp solution for variable Ω S6
S6 Accuracy of the square solution for variable Ω S6

*smi03101@umn.edu

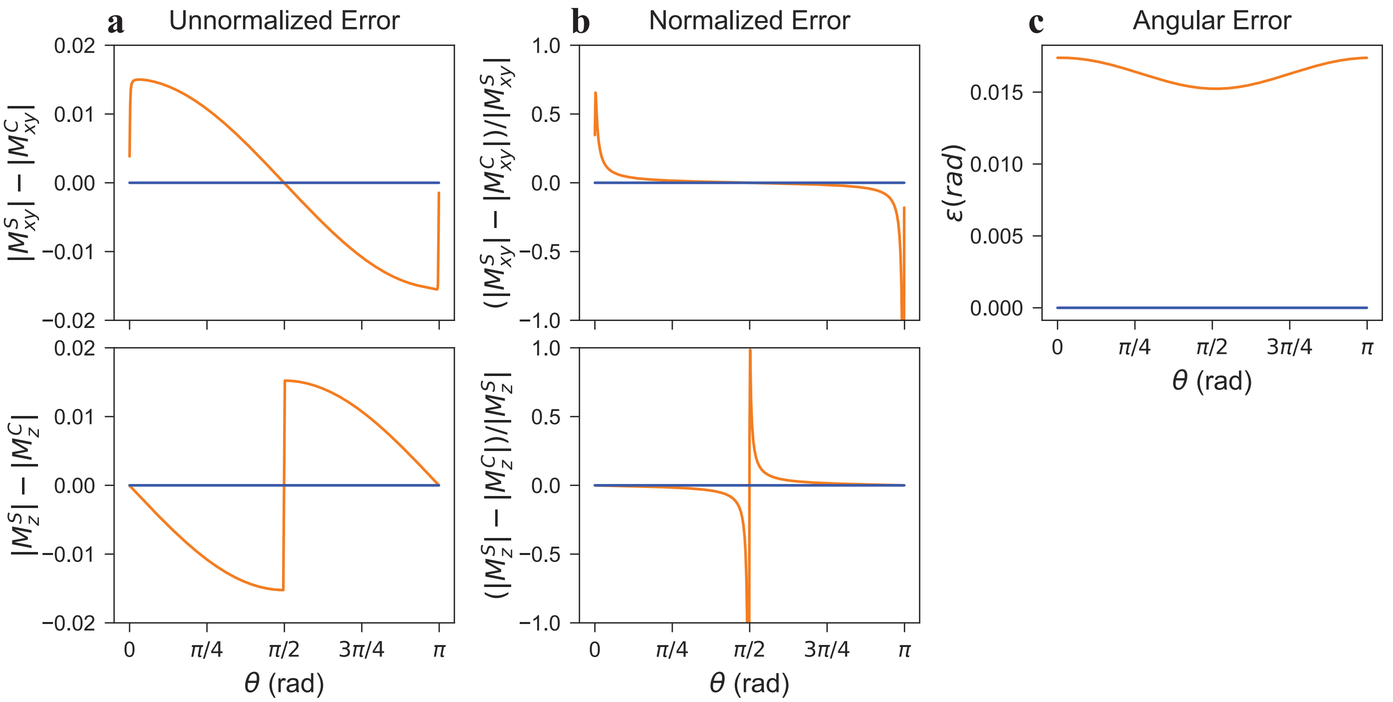

Figure S1: The error of final magnetization between simulation and the method of Zhang et al. (respectively M^S and M^C) shown as (a) unnormalized transverse and longitudinal error, (b) normalized transverse and longitudinal error, and (c) angular error. Initial magnetization orientation was set as $\mathbf{M} = (0, \sin\theta, \cos\theta)$. All calculations and simulations were performed with $\beta = 5.298$, $\omega_1^m/2\pi = 2$ kHz, $T_p = 5.0$ ms, and $R = 8$ with simulations performed over 10,000 time steps. The frequency sweep was intentionally kept low so that final magnet orientations were distributed over a range of elevations to better demonstrate the dependence of both unnormalized and normalized component error on initial (and final) orientation. Component error metrics are well behaved and accurately represent error when the meaningful error is captured in the measured plane or axis but lack the isotropism of angular error.

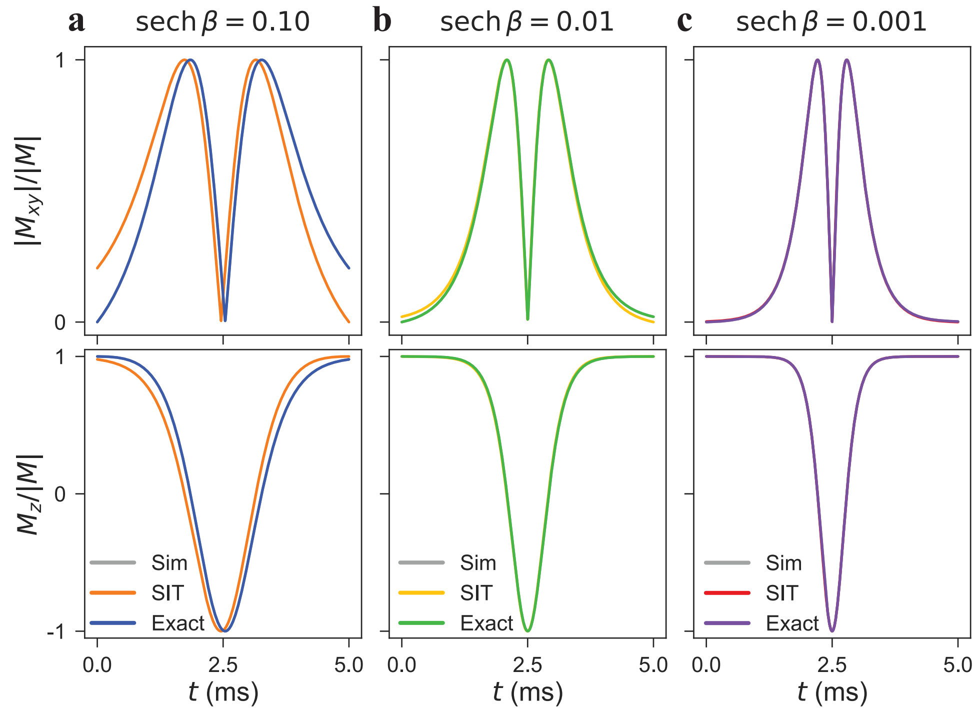

Figure S2: Temporal evolution of the magnetization of on-resonance equilibrium isochromats during HS1 inversion pulses with respective pulse truncations of (a) 0.1, (b) 0.01, and (c) 0.001 as calculated using the exact analytical solution, the SIT prediction, and RK4 numerical simulation. The rows of the columns (a)-(c) respectively show the normalized absolute value of the transverse magnetization and the normalized longitudinal magnetization. ω_1^m was determined in each case by the SIT prediction. Consistent parameters include All results used $T_p = 5.0$ ms and $R = 0$ with simulations run over 10,000 time steps.

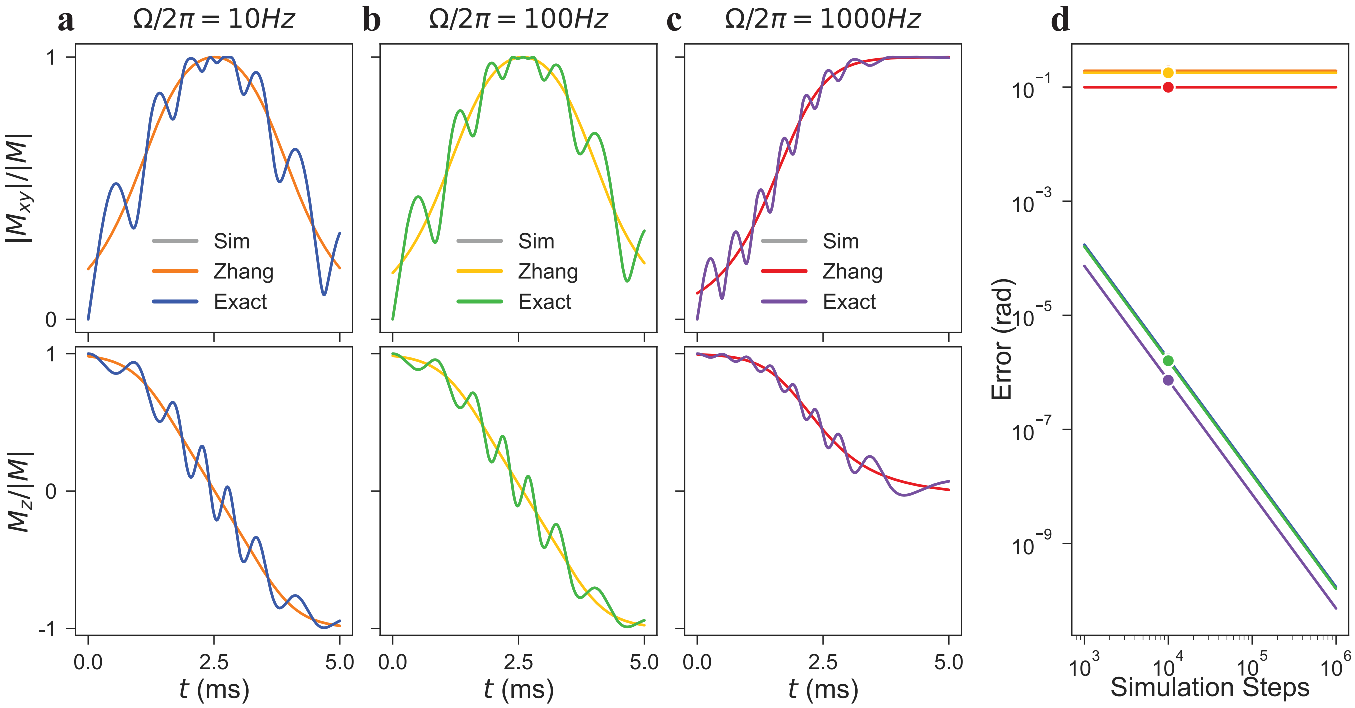

Figure S3: Temporal evolution of the magnetization of equilibrium isochromats during HS1 pulses with $\Omega/2\pi$ respectively (a) 10 Hz, (b) 100 Hz, and (c) 1000 Hz as calculated using the exact analytical solution, the method of Zhang et al., and RK4 numerical simulation. The rows of the columns (a)-(c) respectively show the normalized absolute value of the transverse magnetization and the normalized longitudinal magnetization. (d) The final angular error of analytical solutions relative to simulation results is plotted as a function of simulation time steps. Consistent parameters for these calculations and simulations include $\beta = 2.993$ (i.e. 10% truncation), $\omega_1^m/2\pi = 2$ kHz, $T_p = 5.0$ ms, and $R = 10$ with the simulations of (a)-(c) calculated over 10,000 time steps.

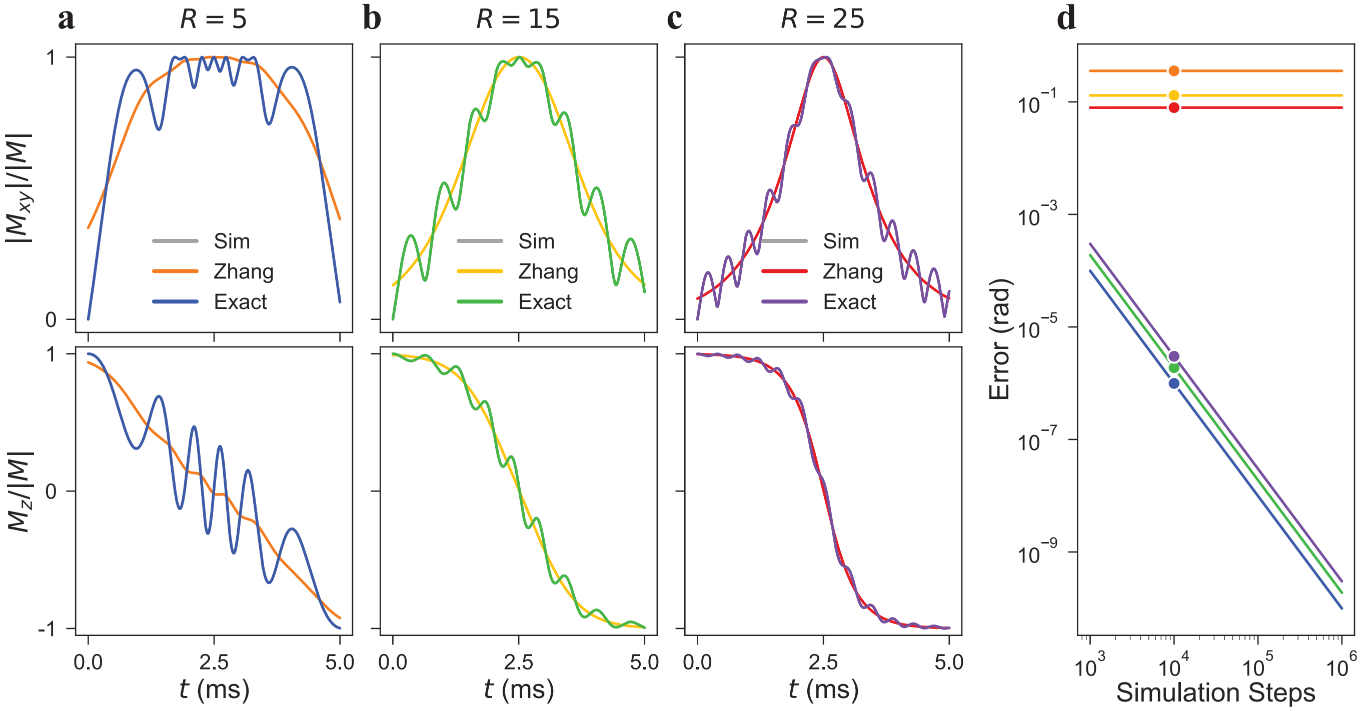

Figure S4: Temporal evolution of the magnetization of equilibrium isochromats during HS1 inversion pulses with R respectively (a) 5, (b) 15, and (c) 25 as calculated using the exact analytical solution, the method of Zhang et al., and RK4 numerical simulation. The rows of the columns (a)-(c) respectively show the normalized absolute value of the transverse magnetization and the normalized longitudinal magnetization. (d) The final angular error of analytical solutions relative to simulation results is plotted as a function of simulation time steps. Consistent parameters for these calculations and simulations include $\beta = 2.993$ (i.e. 10% truncation), $\omega_1^m/2\pi = 2$ kHz, $T_p = 5.0$ ms, and $\Omega/2\pi = 100$ Hz with the simulations of (a)-(c) calculated over 10,000 time steps.

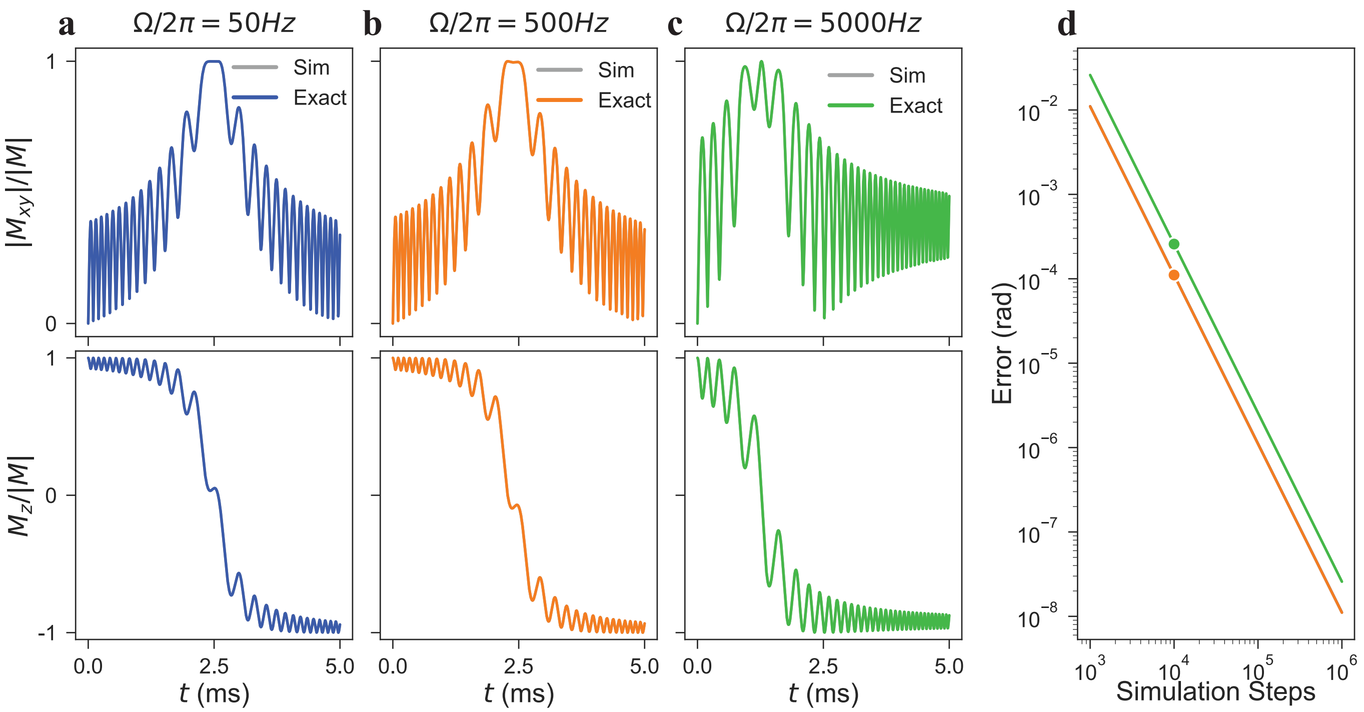

Figure S5: Temporal evolution of the magnetization of equilibrium isochromats during chirp inversion pulses with $\Omega/2\pi$ respectively (a) 50 Hz, (b) 500 Hz, and (c) 5000 Hz as calculated using the exact analytical solution and RK4 numerical simulation. The rows of the columns (a)-(c) respectively show the normalized absolute value of the transverse magnetization and the normalized longitudinal magnetization. (d) The final angular error of analytical solutions relative to simulation results is plotted as a function of simulation time steps. Consistent parameters for these calculations and simulations include $\omega_1^m/2\pi = 2$ kHz, $T_p = 5.0$ ms, and $R = 100$ with the simulations of (a)-(c) calculated over 10,000 time steps.

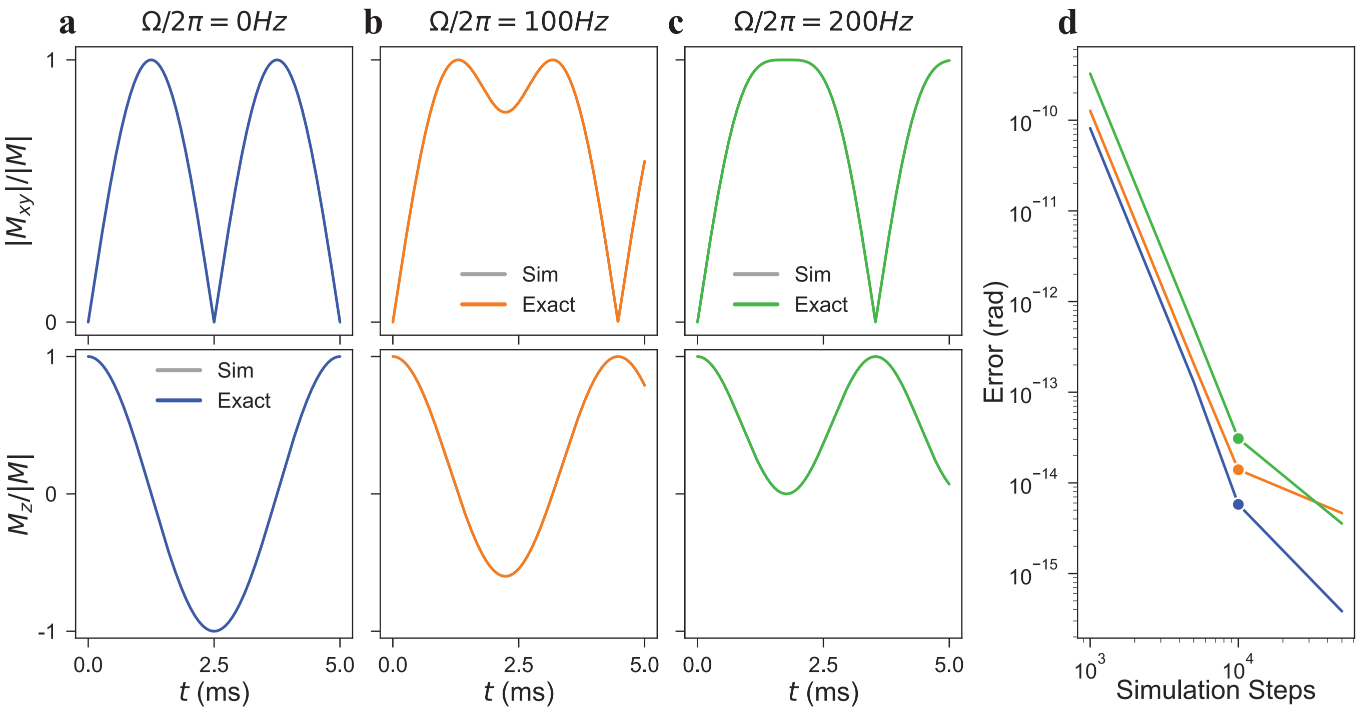

Figure S6: Temporal evolution of the magnetization of equilibrium isochromats during square pulses with $\Omega/2\pi$ respectively (a) 0 Hz, (b) 100 Hz, and (c) 200 Hz as calculated using the exact analytical solution and RK4 numerical simulation. The rows of the columns (a)-(c) respectively show the normalized absolute value of the transverse magnetization and the normalized longitudinal magnetization. (d) The final angular error of analytical solutions relative to simulation results is plotted as a function of simulation time steps. Consistent parameters for these calculations and simulations include $\omega_1^m/2\pi = 200$ Hz, $T_p = 5.0$ ms, and $\phi_c = \pi/2$ with the simulations of (a)-(c) calculated over 10,000 time steps.
